# A Data-Driven Approach to Physical Fatigue Management Using Wearable Sensors to Classify Four Diagnostic Fatigue States

**DOI:** 10.3390/s21196401

**Published:** 2021-09-25

**Authors:** Maria J. Pinto-Bernal, Carlos A. Cifuentes, Oscar Perdomo, Monica Rincón-Roncancio, Marcela Múnera

**Affiliations:** 1 Department of Biomedical Engineering, Colombian School of Engineering Julio Garavito, Bogotá 111166, Colombia; maria.pinto@mail.escuelaing.edu.co (M.J.P.-B.); marcela.munera@escuelaing.edu.co (M.M.); 2 School of Medicine and Health Sciences, Universidad del Rosario, Bogotá 111711, Colombia; oscarj.perdomo@urosario.edu.co; 3 Fundación Cardioinfantil-Instituto de Cardiología, Bogotá 110131, Colombia; mrinron@hotmail.com

**Keywords:** fatigue diagnosis, classification models, inertial measurement units, EMG, walking rehabilitation, physical exercise

## Abstract

Physical exercise contributes to the success of rehabilitation programs and rehabilitation processes assisted through social robots. However, the amount and intensity of exercise needed to obtain positive results are unknown. Several considerations must be kept in mind for its implementation in rehabilitation, as monitoring of patients’ intensity, which is essential to avoid extreme fatigue conditions, may cause physical and physiological complications. The use of machine learning models has been implemented in fatigue management, but is limited in practice due to the lack of understanding of how an individual’s performance deteriorates with fatigue; this can vary based on physical exercise, environment, and the individual’s characteristics. As a first step, this paper lays the foundation for a data analytic approach to managing fatigue in walking tasks. The proposed framework establishes the criteria for a feature and machine learning algorithm selection for fatigue management, classifying four fatigue diagnoses states. Based on the proposed framework and the classifier implemented, the random forest model presented the best performance with an average accuracy of ≥98% and F-score of ≥93%. This model was comprised of ≤16 features. In addition, the prediction performance was analyzed by limiting the sensors used from four IMUs to two or even one IMU with an overall performance of ≥88%.

## 1. Introduction

Exercise rehabilitation during or after medical treatment is considered an effective means of restoring physical and psychological function [1,2,3]. Recent reviews highlight the following benefits provided by physical exercise used as a therapeutic measure. Physical exercise contributes to achieving and maintaining therapeutic goals and improving quality of life, physical function, functional capacities, muscle strength, emotional well-being, and can even reduce depression and anxiety and increase self-esteem [4]. Additionally, it can lower the risk of heart disease, diabetes, cancer, stroke, reduce the risk of orthopedic problems, recover mobility of limbs, strengthen the immune defense (influenza), among others [4,5,6,7]. In this context, physical exercise has the potential to affect people’s health conditions in many ways; hence, the American Health Association established physical exercise as one of the main components of improving people’s health and decreasing morbidity and mortality levels [8,9].

Although these positive health-related outcomes of regular physical exercise are well documented, the amount and intensity of exercise needed to obtain these positive results are unknown [10,11,12]. Several considerations must be kept in mind for its implementation in rehabilitation. For instance, elderly patients have more severe impairments and comorbidities than younger patients; therefore, the rehabilitation needs of the older are different from those of younger patients [1]. The same occurs with individuals with different diseases. Consequently, an essential question when prescribing exercise is what is the optimal therapeutic dose required to produce a specific health benefit according to the individuals’ needs? Typically, when considering exercise dose about health outcomes, exercise is characterized by type, intensity, and volume (session duration and frequency) according to the age, weight, fitness level and pathologies of each patient [13]. Recent studies have shown that intensity is the most relevant feature in prescribing physical exercise [14,15] because it determines the amount of energy expenditure and can be seen as the “dose” of the prescription [16]. Since controlling exercise intensity avoids overtraining patients, which can affect their rehabilitation and make them suffer health consequences (i.e., physical, or physiological complications) [17,18,19]. In this context, it is essential to clarify the meaning of the various terms associated with physical activity and exercise for consistent interpretation of exercise intensity in the context of dose–response issues.

Intensity is the magnitude of the increase in energy expenditure necessary to perform the activity (aerobic or endurance exercise) or the force produced by the muscle contractions (resistance or strength exercise) [20,21]. Thus, the intensity has been used to classify the physical exercise into three groups: low-intensity exercises that are composed of soft activities which demand low energy cost (50% of the maximal HR (HR${}_{max}$)), and are usually used for patients with extreme risk conditions; moderate-intensity exercises that contemplate non-stop activities with a long duration that require a low or moderate effort around 50% to 75% of the patient’s HR${}_{max}$; and high-intensity exercises that are workouts that alternate hard-charging intervals with a short duration (15 s to 5 min) that increase the HR${}_{max}$ up-to 85% to 100% with a recovery period of equal or longer duration than the work interval [16,22]. Regarding prescribing exercise, moderate-intensity training is the most implemented in the rehabilitation process because it involves large muscle groups in dynamic activities that result in substantial increases in HR and energy expenditure [23,24]. The main challenge for the implementation of these exercises is the difficulties in managing the intensity, which makes their prescription a complex task [19,22,23].

Some successful rehabilitation programs have been explored to supervise the exercise intensity during therapies through monitoring of the patient’s fatigue state [1,21,24,25,26]. Fatigue has generally been defined as a subjective state of tiredness or exhaustion and the reduction of capacity for regular activity [27]. Additionally, it is defined as the inability of the muscles to maintain the required level of strength during exercises. It can also result in the deterioration of health in the long term, including work-related musculoskeletal disorders [28], chronic fatigue syndrome [29] and compromised immune function [30]. Therefore, fatigue is a common concern among clinicians and individuals who participate in physical activities based on training or rehabilitation [27]. An important first step in managing fatigue is the rapid and accurate detection of its occurrences. However, because of the wide range of factors that can produce fatigue, there is no scientifically accepted method to identify it.

## 2. Related Works

Diverse fatigue detection techniques have been studied and used in rehabilitation that can be divided into qualitative and quantitative approaches. Qualitative methods are centered around the use of subjective scales of fatigue perception [31,32,33]. Some questionnaires consist of asking patients about their perceived tiredness level according to a pre-established ordinal numeric scale [34,35]. One of the most implemented in physical rehabilitation is the ten points Borg Rating of Perceived Exertion scale (Borg CR10) where a lower number represents a state of absence of fatigue, and a higher number represents a state of extreme fatigue [36]. However, the understanding of how an individual’s performance changes throughout rehabilitation is limited because the qualitative methods do not always represent the actual intensity has led to a decrease in reliability [32,37,38].

Quantitative approaches such as physiological parameters and exercise performance have been proposed for continuous monitoring of patients with chronic diseases during physical sessions [29,39,40,41,42]. Regarding physiological parameter measurement, one of their applications is the indirect estimation of fatigue [30,43,44,45]. The parameters most related to fatigue are: oxygen uptake (VO${}_{2}$) that represents the oxygen consumption that the body takes up and utilizes the exercising muscle [46]; heart rate (HR), which is one of the most used physiological parameters to control fatigue [47,48] due to its measurement facility and the linear relationship with VO${}_{2}$ [49]; and blood lactate, which is one of the most often measured parameters during clinical exercise testing as well as during performance testing of athletes [50]. It is essential for clinicians to understand the pathological response as well as the normal response to exertion [51]. However, it requires a blood sample and a specialized instrument, which are not easy to get during physical therapies [52]. Although the physiological parameters are considered accurate in measurement technique terms, these parameters present difficulties to monitor in real-time due to their acquisition processes. In addition, they may present different behaviors depending on the exercise type performed (e.g., moderate or high-intensity exercise), which makes it difficult to relate them to the fatigue level.

Considering that regardless of the exercise type performed the exercise performance has a directly proportional relationship with fatigue [53], several methods for monitoring fatigue through exercise performance have been implemented using ambulatory sensors (e.g., electromyography and inertial sensors) or non-ambulatory sensors (e.g., motion analysis system) to identify when an event exists outside the typical pattern, which supports the rehabilitation process and performance monitoring of activities [54].

Electromyography (EMG) is considered the gold standard to detect muscular fatigue considering that it directly assesses the bio-electrical muscle function [55,56]. However, EMG processing is a complex task to execute in real-time, since it requires power and frequency analysis [57] to identify fatigue progression. Therefore it inhibits their daily usage for real-time fatigue detection. Inertial measurement units (IMUs) are reliable and cheap sensors that are used to capture a person’s acceleration and motion data in real scenarios without the use of external sources or devices [58,59,60], allowing to assess the activity performance through estimations of the kinematic and spatiotemporal parameters [61], and motion analysis [62]. Although it is possible to identify the person’s fatigue level using these sensors, considering that the kinematic study in fatigue is still an early topic, the use of other physiological parameters like blood lactate [63], EMG [64], or even perceived level of fatigue [65] are widely used to corroborate the results. A motion analysis system is widely used in fatigue estimation due to its high accuracy and robustness in the measurement of the kinematic parameters. The motion analysis system is based on the use of infrared cameras to estimate the position of reflective markers to segment an object or an individual and measure variables such as position and orientation [66]. However, motion capture systems often require special setups which make them better suited for controlled environments [67].

As this article has already pointed out, humans’ performance changes as a function of a person’s individual characteristics (e.g., age, gender, fitness level, injury history, etc.), time (which can be manifested through detrimental performance due to fatigue and improved performance due to learning effects) and degree of exercise difficulty. Therefore, to enhance fatigue estimation the use of artificial intelligence systems in optimizing and transforming human performance has been implemented as a further alternative to monitor and understand how an individual’s performance deteriorates with fatigue accumulation [38,68]. These models appear as a complementary alternative to collected human performance data from the diverse detection techniques (i.e., qualitative or quantitative approaches) that allow classifying fatigue levels. Table 1 summarizes fatigue modeling research using artificial intelligence systems. These studies differ between the physical activity movement performed, the sensors used, and the fatigue identification. For instance, some studies attempted to identify high/extreme fatigue that results in an inability to generate muscle forces and consequently, an individual’s performance decrease and inability to perform the physical activity [69,70,71,72]. The other studies focused on detecting fatigue without reaching exhaustion, where individuals are still able to perform their physical activity at a diminished level [38,73,74,75]. Since exhaustion in physical activity is often localized, the associated literature [69,70,71,72,73,75,76] focused on one physical activity only (e.g., walking or squats) primarily utilized 3D motion capture systems, EMG, accelerometers or IMUs, which were used to model the individual’s performance, whereas only two studies developed models to focus on a more complex task [38,74], which utilized IMUs and HR monitors. Five studies used a subjective fatigue scale as a reference of the individuals’ fatigue perception in contrast to their implemented fatigue detection method [38,70,73,74].

These models presented a significant potential for clinical scenarios because they provide an objective indicator of the user’s fatigue condition. However, they considered only two fatigue states, i.e., fatigued, or non-fatigued state, which were generally obtained in two separate steps; in the first step, the participants have no fatigue while in the second step fatigue is induced to the participants. The above limits the accurate monitoring of the user’s exhaustion during therapy, restricts the possibility to determine the adequate “dose” (i.e., intensity) of the individuals to produce a specific health benefit according to their individuals’ needs, and thus limits improvements to the user’s performance during therapy. On the other hand, from a detailed literature review, we could not identify any article that discussed or established the best classifier for the identification and diagnosis of fatigue. This may be attributed to the lack of understanding of how an individual’s performance deteriorates with fatigue accumulation, which can vary based on user conditions and physical activity.

As a first step, this paper proposes a framework for developing a fatigue identification model based on the individuals’ exercise performance assessment to classify four fatigue diagnosis stages (low, medium, high, and very high). The fatigue diagnosis stages allow clinicians to pinpoint the hazard directly. They can then prescribe interventions from a large number of options, including assigning rest breaks (which can reduce the level of fatigue before it reaches potentially dangerous levels) or redesigning the activity (which can eliminate the development of fatigue) [38]. Four fatigue stages allow accurate monitoring of patients’ fatigue conditions during exercise to avoid any injuries or affect the rehabilitation process.

To this end, our framework takes benefits from the advances and widespread use of wearable sensors for data collection. These sensors offer an individualized insight into the individuals’ performance and present a unified performance benchmark that does not depend on process cycle time (it is essential for real-time scenarios). Furthermore, we have chosen to focus on monitoring fatigue in walking tasks since (i) localized muscle fatigue is a potential risk factor for injury or falls as muscle fatigue adversely affects proprioception, movement coordination and muscle reaction times, leading to postural instability and gait alterations [65,69,73,76,77]; therefore, gait patterns associated with fatigue may help in the assessment of fatigue-related fall risks or injuries in various environments; and (ii) moderate-intensity training as walking exercises are one of the most used in physical rehabilitation process due to improving cardiovascular system and skeletal muscle function [20,22,23,78].

In this context, this framework allows to understand how an individual’s performance deteriorates with fatigue accumulation in walking tasks. The code and data implemented in this work are offered as supplementary materials to encourage adoption in practice and further investigations by researchers.

Finally, it is significant to emphasize that social interaction has been shown to have a positive impact on general mental and physical well-being in physical exercise therapy [79,80]. Therefore, the use of these artificial intelligence systems has a great potential in physical rehabilitation scenarios application through the use of socially assistive robots (SAR) [81,82,83]. SAR is a system that employs interaction strategies, including the use of speech, facial expressions, and communicative gestures, to assist according to a specific healthcare environment [80]. Several studies have shown that SAR systems improve (i) the ability to influence the patient’s intrinsic motivation to perform the task and (ii) the ability to personalize the social interaction to maintain user engagement in the task through permanent patient monitoring and feedback [79,81,82,83,84,85,86]. Therefore, with the support of the machine learning models, this technology has been used to provide better service and improve patient’s experience and treatment outcomes [81,83]. An example of the above is the study conducted by Casas et al. [81], who implemented a social robot to act as a training assistant in a cardiac rehabilitation program in a clinical setting. The results suggested that SARs are well received by patients and have a positive impact on their willingness to perform prescribed rehabilitation exercises. In summary, the social robot achieved continuous monitoring and continuous feedback to patients on their performance, leading to an increase in their motivation during the rehabilitation session. Similar outcomes are reported by [79,83]. In this regard and the context of fatigue detection, a future better application would be to implement these machine learning models in the RAS. Because this would provide continuous monitoring and feedback on the patient’s performance, allowing to control the intensity and the correct execution of the exercise. Similarly, alerts could be generated when the patient is presenting high levels of fatigue or there is a variation in exercise performance parameters. This is very promising for clinical scenarios and would be of great help to avoid overtraining in patients, which could affect their rehabilitation and even suffer physical or physiological complications.

## 3. Materials and Methods

The study has been divided into two parts: an experimental setup to obtain the corresponding dataset and a proposed framework for a fatigue classifier to develop and assess the fatigue estimation models. Specifically, six classification strategies have been proposed and implemented to automatically identify four fatigue diagnosis levels in walking exercises by monitoring 43 kinematic/temporal and biomechanical features.

### 3.1. Experimental Setup

The experimental protocol was performed to quantify the detection success ratio of the fatigue classifiers. A total of 24 healthy subjects (14 males, 10 females, 21.75 ± 1.16 years old, 1.71 ± 0.09 m) performed the study, who perform regular physical exercise and who had no known physical or cognitive disability, injuries, pain, or any impediment to exercise (see Table 2 for further information). The participants enrolled in this study, must not present any fatigue state, i.e., they had to be in a non-fatigued state to avoid affecting their test performance. To this end, five different types of fatigue—general fatigue, physical fatigue, mental fatigue, reduced motivation, and reduced activity—were assessed using the “multidimensional fatigue inventory” [87] questionnaire. All subjects presented non-fatigued conditions, and were informed about the scope and purpose of the experiment. Written consent was obtained from each of them before the study. The Ethics Committee of the Colombian School of Engineering Julio Garavito, Bogota, Colombia approved the protocol.

**Figure 1:**
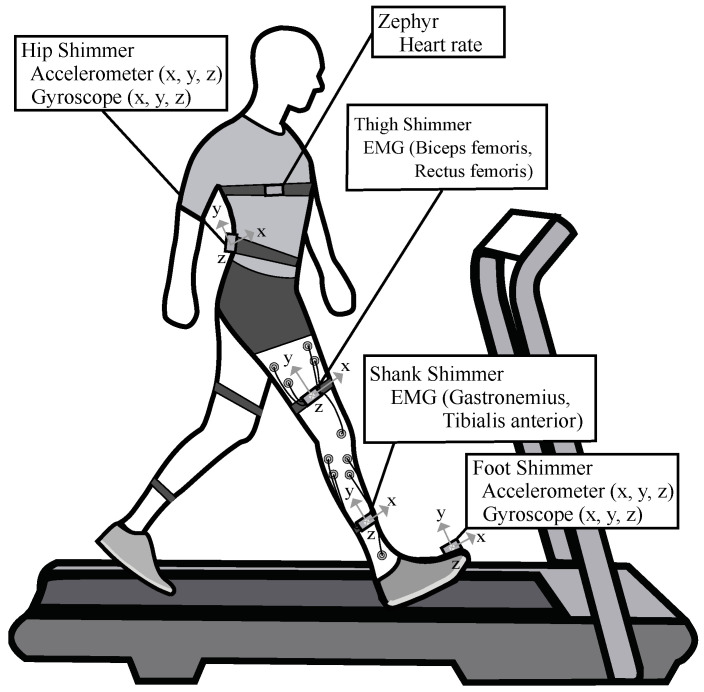
Experimental setup. Each participant was instrumented on their dominant side with an IMU placed on the dorsal side of their foot and with an IMU placed on their spine between L2 and S1. two more IMUS located on each thigh and shank with different electrodes situated in tibialis anterior, rectus femoris, biceps femoris, and gastrocnemius. Participant’s heart rate was captured using the Zephyr sensor located on their chest.

Volunteers were first instructed to perform three 10 m tests at a self-selected speed to determine their average overground speed, which was successively set on a treadmill (NIZA RX K153D-A-3, SportFitness, Bogota, Colombia). Participants were equipped with four Shimmer3 (Shimmer, Dublin, Ireland) IMU units, one located on their foot dominant instep and one located around the center of L5-S1 with a sample rate of 128 Hz. The remaining two units were configured with a sampling rate of 512 Hz to obtain EMG signals in four muscles (tibialis anterior, rectus femoris, biceps femoris, and gastrocnemius) of the participant’s dominant leg. One IMU was located on the outer lateral part of the thighs and one on the calves’ outer side with elastic bands, the EMG signal was recorded from a pair of Ag–AgCl electrodes (interelectrode distance 3 cm) after cleansing the skin by alcohol. In addition, participants were equipped with a Zephyr HxM BT (Medtronic, Ireland) on their chest with an elastic band, with a sample rate of 1 Hz. The selection of the Zephyr BT sensor is based on accuracy, reliability, cost, availability, and comfort [88,89]. The experimental setup described is illustrated in Figure 1.

Participants were then asked to walk for at least 120 s on the already configured treadmill at a zero-degree inclination, where different parameters were assessed. The first two parameters directly indicate the fatigue level: blood lactate and a perceptive fatigue scale using the Borg CR10. Therefore, they were used as reference values to diagnose and classify the participants’ fatigue level. At the same time, kinematic/spatiotemporal parameters, and EMG signals were recorded. Afterward, participants’ fatigue inducement was carried out. Volunteers had to perform as fast as possible a physical exercise circuit composed of four exercises: high knees, jumping jacks, squats, and short runs. At the end of the physical exercise circuit the participants returned to the treadmill and the whole process was repeated.

The above was repeated four times to increase the participants’ fatigue level. The difference between each round was the execution time of the physical circuit that increased progressively. In other words, the time corresponding to the performance of each exercise increased by 15 s each round; for the first time, each exercise was performed for 30 s, the second round for 45 s, the third round for 60 s, and the last round for 75 s. Note that if the participants’ HR overcame 90% of the HR${}_{max}$, or a 10 Borg value was notified, the test was immediately concluded. The entire experiment, including donning/doffing times related to instrumentation procedures and walking tasks, was completed within 60 min for all volunteers.

It is worth highlighting that to get an approximation of each volunteer’s HR${}_{max}$, Tanaka’s formula using the user’s age (in years) was implemented as is shown in Equation (1). Tanaka’s equation is recommended for healthy individuals such as those involved in this study because this equation significantly overpredicts maximal HR. Therefore, for people who present some diseases, it is recommendable to adapt this method when using exercise testing [90].

(1) $$HR_{max}=206.9-\left(0.7*age\right)$$

Regarding the measured parameters, they were measured every time that participant returned to the treadmill. The blood lactate sample was taken from the participant’s earlobe with a new lancet. The blood was collected with a new test strip, and finally, the strip was inserted into the Lactate Pro2 (Arkray, Japan) to measure the blood lactate level. On the other hand, the Borg CR10 scale was obtained by asking the volunteer how tired they felt according to the scale; Table 3 was used to explain the values meaning to each volunteer according to the four fatigue levels (low, moderate, high, very high). The participant was also instructed only to focus on the total effort sensation and not on shortness of breath or muscle pain. Concerning the data acquisition, it only started once the self-selected speed was reached, and the treadmill speed was only reduced after all data were acquired to prevent data capture during the transient state.

### 3.2. Proposed Approach for Fatigue Classifier

**Figure 2:**
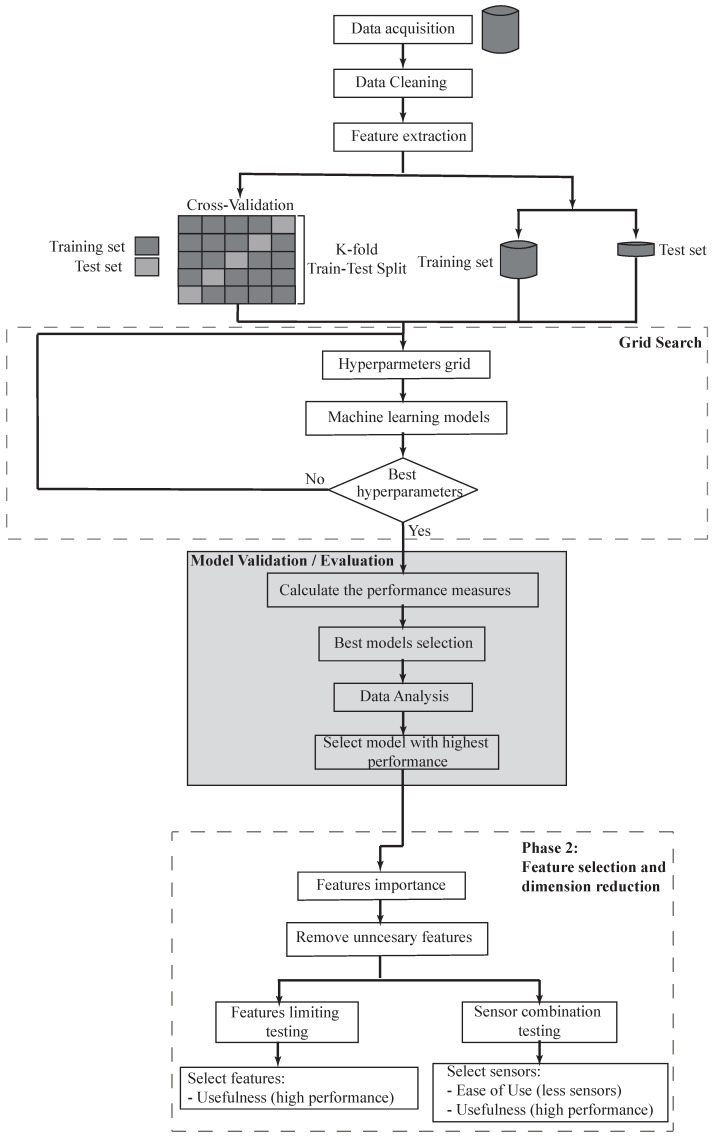
Flowchart that illustrates an overview of proposed method.

Figure 2 presents an overview of the proposed framework for managing physical fatigue. This framework was divided into two phases. The first phase consisted of the selection of an appropriate classifier model for prospective analysis. To this end, the first phase is comprised of three main steps: (i) data preprocessing and feature generation, where the sensors’ data are prepared for analysis and generated the dataset; (ii) model construction and validation, where statistical and data analytic models are trained for distinguishing between the four fatigue states (non-fatigued, low-fatigue, moderate-fatigue, high-fatigue); (iii) data analysis, where the classifier models are evaluated based on accuracy, precision, recall and F-score. In the second phase, where the best model is established, the subset of features/predictors that are most frequently used in predicting the fatigue state are identified.

### 3.3. Phase 1: Fatigue Detection

#### 3.3.1. Data Preprocessing

**Figure 3:**
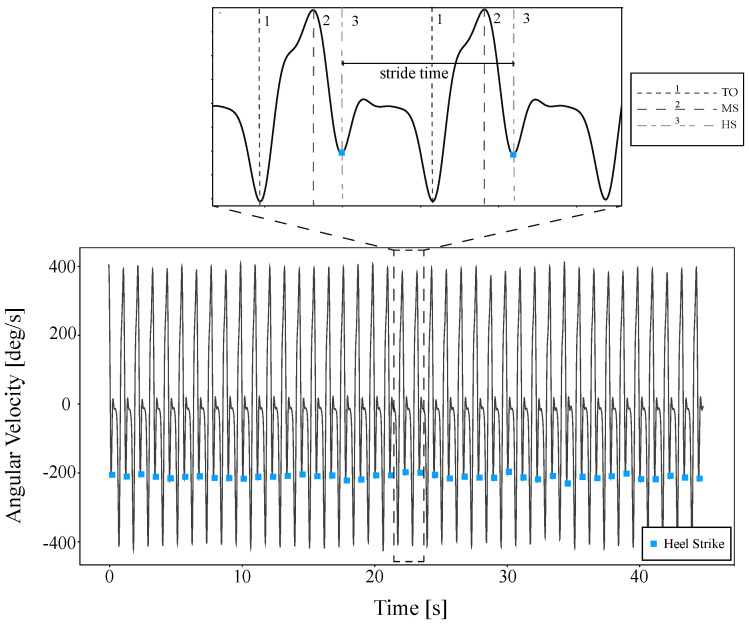
Heel strike detection using an inertial detection system on a participant’s test record. The zoom part represents two gait cycles and the identification of each gait phase: TO = Toe-Off (first dashed line), MS = Mid-Swing (second dashed line), and HS = Heel Strike (third dashed line).

The first step in analyzing data is to ensure that the data is correct and cleaned. Therefore, the fourth main cleaning step was proposed. First, the gyroscope and accelerometer outputs were treated with a second-order low-pass filter Butterworth for noise removal [91,92]. Second, the collected data were visualized to check any additional erroneous data (e.g., faulty sensor values (too high and too low)), i.e., data that were not corrected through the automated filtering in the previous step. Third, the data from the different sensors were synchronized and any observations that were captured outside of the experimental window were eliminated. Fourth, the data were partitioned using a non-overlapping time window, where the selection of the length of the time window depended on the identification of each participant’s gait event. Considering that gait presents a repetitive behavior, an automated procedure was implemented to detect each participant’s gait cycle. Specifically, the process consisted of determining each heel-strike event by detecting the second minimum value in the angular velocity signals of each stride time. This process outcome is represented in Figure 3, where a sample of each heel strike of the corresponding signal from a participant’s test record can be observed. In addition, a zoom of a signal part is presented in Figure 3, where gait event detection throughout the angular velocity of an inertial sensing system over two gait cycles can be appreciated [60]. The selection of the correct threshold value was carried out as in [93], whose study validated all possible thresholds of the gait cycle within a range, and whose limits were visually established from signals acquired in a preliminary analysis.

Regarding feature generation, two IMUs were attached at the participants’ foot instep and around L5-S1 to measure the acceleration associated with a person’s dynamic motion (spatiotemporal and kinematic parameters). These features captured the intensity and spread, which are commonly used in the fatigue detection literature [74,94,95]. Likewise, two more Shimmer3s were used and configured to obtain EMG signals in four muscles (tibialis anterior, rectus femoris, biceps femoris, and gastrocnemius) to register electrical potentials [96]. These potentials are directly related to muscular strength, which allows to estimate the effort and evaluate the participants’ exercise performance [97]. The description of the proposed features is provided in Table 4. Note that these features are calculated for each time window, i.e., for each gait cycle.

It is essential to highlight that feature variability caused by each participant’s physical condition makes it difficult to perform a direct comparison among the volunteer registers, which requires a normalization of the data according to each initial subject performance [104,105,106]. All features extracted were normalized by dividing them with the corresponding initial value (see Equation (2)). Note that the test number zero of each volunteer was taken as a reference since it was considered that the volunteer did not have fatigue, which was corroborated with the blood lactate, the Borg CR10 scale, and the multidimensional fatigue inventory questionnaire.

(2) $$F_{i}=\left(\frac{F_{i}}{F_{reference}}\right)$$

where $F_{i}$ corresponded to each feature extracted in each time window and $F_{reference}$ is the average of the first ten time window values of the test number zero where volunteers were not fatigued.

#### 3.3.2. Model Construction and Validation

In machine learning, there are several methods of data partitioning for experimentation. The most popular methods are typically referred to as training/test partitioning or cross-validation [107,108]; both were implemented in this research to evaluate the best classifier performance.

The training/test partitioning typically involves the partitioning of the data into a training set and a test set in a specific ratio, e.g., 70% of the data are used as the training set and 30% of the data are used as the test set. This data partitioning can be done randomly or fixed. The fixed way is typically avoided (except when order matters) as it may introduce systematic differences between the training set and the test set, which leads to sampling representativeness issues. To avoid such systematic differences, the random assignment of instances into training and test sets are typically used [108,109].

Cross-validation is conducted by partitioning a data set into n folds (or subsets), followed by an iterative process of combining the folds into different training and test sets. In other words, each of the n folds is, in turn, used as the test set at one of the n iterations, while the rest of the folds are combined as the training set [110]. A typical approach to cross-validation is dividing the dataset into 10 folds, where the models are selected based on the average/median prediction performance across 10 non-overlapping test datasets. The literature suggests that 10-fold cross-validation may reduce the variation between the training and test performance [111]. However, cross-validation is generally more expensive in terms of computational cost than training/test partitioning.

Regarding the selection of the machine learning models, several classification methods are viable candidates for utilization in fatigue prediction. However, from our framework’s perspective, it is impossible to predetermine which methods will work best for fatigue prediction in the walking task. Because these methods are data-driven and thus, are application-dependent, i.e., dependent on the exercise, extracted features, sensors, scenarios, among others, several methods were applied during our preliminary analysis of the data to develop the fatigue prediction model. The models evaluated included: logistic regression (LR), decision trees (DT), k-nearest neighbors (KNN), support vector machine (SVM), naive Bayes (NB), linear discriminant (LDA), artificial neuronal network (ANN), and random forest (RF). The open-source python library “scikit-learn” [112] was used to execute a quick general training for these classifiers. Afterward, according to the accuracy metric and due to their relatively poor performance, LDA and NB were eliminated. Hence, our case study focused on using the best six classification models (LR, KNN, SVM, RF, ANN, and DT), adjusted and retrained, by modifying their training hyperparameter automatically through computational iterations. For a detailed introduction on the classifier mentioned above, the reader is referred to [113,114].

#### 3.3.3. Data Analysis

Developments in machine learning classifiers from imbalanced data have been mainly motivated by numerous real-life applications since it faces the problem of unequal representation of the data [115]. Most of the machine learning models used for classification have been designed around the assumption of an equal examples distribution for each class [116]. This means that an incorrect application of a model may focus on learning the characteristics of the abundant observations only, neglecting the examples from the minority class increasing false positives. However, a slight imbalance, i.e., the distribution of examples is uneven by a small amount in the training dataset (e.g., 4:6), is often not a concern [117,118]. In this context, to evaluate the performance of the proposed fatigue detection models, it was essential to consider the following performance measures.

Recall or true positive rate (TPR), which captures the ability to detect the fatigued cases, i.e., quantifies the number of positive class predictions made of all positive examples in the dataset, is computed as follows

(3) $$Recall=\frac{True\;Positive}{True\;Positive+False\;Negative}$$

where a true positive (*TP*) is considered if the classifier prediction and the reference value match. Otherwise, such classification is regarded a false positive (*FP*), i.e., it is the number of registers that belongs to other groups and were wrongly estimated. Likewise, accuracy presents the percentage of correct classifications made by a given model (Equation (4), where *n* represents the complete amount of data).

(4) $$Accuracy=\frac{1}{n}\left(\frac{TP+TN}{TP+FN+FP+FN}\right)$$

where the non-fatigue state is similarly detected by a classifier and reference signal correspond to true negative (*TN*); otherwise, they have been accounted for by false negatives (*FN*). Precision, which quantifies the number of positive class predictions that actually belongs to the positive class, was measured as follows

(5) $$Precision=\frac{TP}{TP+FP}$$

The last metric considered was F-score (Equation (6)) which provides a single score that balances both the concerns of precision and recall in one number.

(6) $$F1_{micro}score=\frac{Precision*Recall}{Precision+Recall}*2$$

### 3.4. Phase 2: Feature Selection and Dimension Reduction

Once the best prediction model is identified, it is essential to consider that a critical aspect for technology adoption is usability. When the number of potential features/predictors is large, the computational complexity for model training increases. Feature reduction is typically applied to reduce the computational burden. In general, models are more interpretable if the number of features is smaller, which could (i) equalize or even increase the performance metrics of the classifier by removing unnecessary features from the data, and (ii) increase the generalization capability [119]. In the context of our framework, usability can be measured using a total number of features selected; therefore, we hypothesize that the chosen prediction model will have a relatively low number of features. Considering that the proposed framework enables the diagnosis of fatigue and the recommendation of an appropriate intervention, the feature selection/reduction was performed through univariate statistical approaches where the feature selection was based on their relationship to the response and their prediction performance. From this step, practitioners can understand which features affect fatigue and how they are associated with changes in the potential classifier. Therefore, any unchanged features in the fatigued and non-fatigued states should be removed. This suggests that the result would be a more interpretable fatigue classifier with relatively large prediction performance, i.e., a good fatigue detection classifier with a low false alarm rate.

## 4. Results

Table 5 summarizes the samples numbers of the four fatigue states. The “Low” class presents most of the registers with 43%, followed by the “Moderate” with and “High” classes with similar proportions (26.41% and 25.08% ). The “Very High” class has the lowest value, with a difference of 10% between the “Moderate” and “High” classes, and a difference of 16.70% regarding the “Low” class. The difference between the classes represents an imbalanced dataset.

The predictive performance of the six models is summarized in Table 6. The table shows the mean for each of four metrics. The reported results are based on 2919 dataset samples from the respective partitioning method: training/test (i.e., 80% of the data were used as the training set and 20% of the data was used as a test set). For the first three numeric columns, a higher value is desired since it reflects a better prediction performance. Note that since the data set obtained is imbalanced, which causes an increase of the false positives, the F-score was considered as the most relevant feature for the performance classifier selection. Moreover, Table 6 reports the parameters and the performance of the six classifiers implemented. The logistic regression (LR) classifier implements the large-scale bound-constrained optimization as a penalty algorithm (solver = newton-cg), and a value of 1,000,000 for its inverse of the regularization strength learning parameter (C = 1,000,000). The k-nearest neighbor (KNN) method using Euclidean distance classified the registers by a majority vote of its nearest elements with three neighbors (K = 3). The decision tree (DT) method using the function to ensure the quality of a split (criterion = entropy) and the tree depth to control the size of the tree to prevent overfitting (max depth = 12) can create arbitrarily small leaves (min samples split = 11) and guarantees that each leaf has a minimum size (min samples leaf = 4), avoiding low-variance over-fit leaf nodes in regression problems. The support vector machine (SVM) has a radial basis function kernel (kernel = balanced) and a constrain value of 64 (C = 64). The artificial neuronal network has a stochastic gradient-based optimizer (solver = adam), 100, 100, and 100 as hidden layer sizes (HLS = (100, 100, 100)), activation function for the hidden layer (activation = tanh), learning rate schedule for weight updates (learning rate = constant), and regulation term parameter (alpha = 0.0001). Finally, the best model is a random forest classifier with 100 estimators (n estimators = 100), which means that the model integrates 100 decision tree models to merge their prediction. Note that all the hyperparameters used for the generation of the classifiers are presented in Table 6, allowing for their easy replication.

The next step is to analyze how the prediction performance varies while limiting the number of features or the number of sensors used in the study. The random forest method was selected to analyze the effect of removing features according to the highest performance metric reported in Table 6. The results of this approach are presented in Table 7, with (i) feature reduction using all sensors, and (ii) where features are limited to those from one and two sensor combinations.

**Figure 4:**
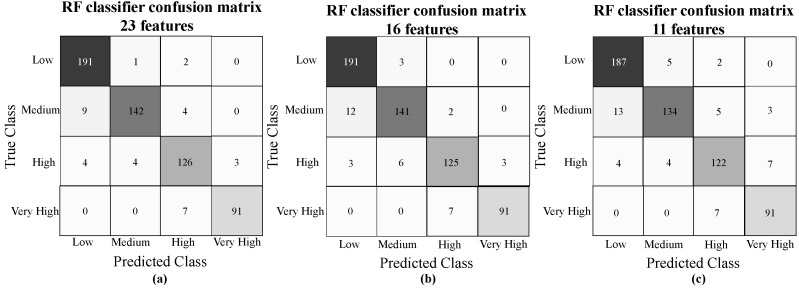
RF classifier confusion matrix for (**a**) 23 features, (**b**) 13 features (**c**) 11 features.

The confusion matrix obtained from the best three RF classifiers models with feature reduction implemented after exploring in a grid search manner is shown in Figure 4, where along the x-axis are listed the true class labels and along the y-axis are the class prediction. Along the first diagonal are the correct classifications, whereas all the other entries show misclassifications.

**Figure 5:**
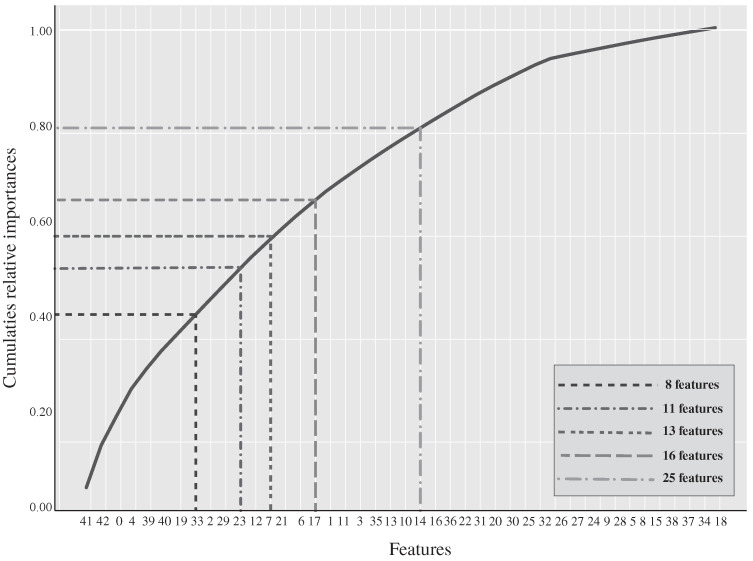
Features relative importance for random forest classifier using the original train data.

Regarding the feature reduction used, it was essential to consider the feature importance properties for the initial RF model. The feature importance property measures a relative weight value to each feature, which represents a direct relationship with the importance of the corresponding feature for this classical machine learning model. Figure 5 presents a cumulative graph which represents the relative importance values obtained for each feature, sorted from the highest to the lowest values.

## 5. Discussion

The determination of the four fatigue states was achieved using qualitative methods such as the Borg scale, as well as quantitative methods using blood lactate measurement. This last parameter was the most considered and used to measure the performance and fatigue of individuals. This is because in response to progressively increasing exercise, lactate will increase exponentially. An individual’s endurance performance is well correlated with their blood lactate [50]; therefore, lactate monitoring increases the confidence of healthcare personnel in assessing the patient’s effort in physical therapies [51]—in other words, blood lactate is a direct indicator of fatigue. This, in turn, makes the fatigue classification model more accurate and reliable, since the data delivered by the sensors are directly related to what is happening physiologically with the user.

The heart rate was not considered as a feature in the classification models considering that this variable is more related to controlling the intensity of the exercise rather than determining the individual’s physical fatigue (i.e., it is not directly proportional to the individual’s fatigue state) [49]. For instance, given a scenario where the individual is exercising at a constant speed, the HR may remain stable and the individual may be experiencing a level of fatigue which is not reflected in the measurement, and vice versa. The second reason for not considering HR was bearing in mind that it is determined by different variables such as age, the physical condition of the individual, comorbidities, and gender; therefore, when standardizing this variable it may cause a degree of uncertainty [47,48].

According to the dataset distribution presented in Table 5, the main difference between classes was 16.79%, which is considered as a slight imbalance, and it is acceptable for data analysis and training computational models [117,118]. Considering the report by Fernandez et al. [116] that a slight imbalance can often be treated like a normal classification predictive modeling problem as long as true negatives are considered, the selection of the best model was performed mainly based on the F-score.

The fourth main observations from the predictive performance of our six models presented in Table 6 need to be highlighted. First, as expected from the preliminary analysis, the classifiers implemented with training/test presented a higher performance in all metrics than the classifiers implemented with cross-validation. Hence, a simple training/test split is sufficient for larger datasets. Second, the performance of the five models, except for the LR classifier, is relatively high with an overall average F-score greater than 80.4%. Third, according to the literature review, it was expected that the LR classifier presented a better performance given the positive results obtained with this classifier in [38,73,74]. However, this model presented the lowest performance with a 62% F-score, which would not represent an optimal or good classifier. This can be associated with two main factors: (i) These studies considered only two fatigue conditions: fatigue or non-fatigued, whereas this work contemplated four fatigue states. It increased the probability of failing in the estimation and suggested that this classifier performed better for binary classifications problems. (ii) Regarding the lack of understanding of how an individual’s performance deteriorates with fatigue accumulation, which can vary based on user conditions, physical activity, features extracted, and fatigue detection technique, it is essential to have a general framework for fatigue estimation classifiers as presented in Section 3.2 to guide the implementation, evaluation, and continuous improvement of fatigue monitoring in rehabilitation scenarios regardless of physical activity or user conditions. Fourth, the RF model presented the best performance in all features with an overall of 92% considered as an optimum classifier for the fourth state estimation of fatigue in walking tasks.

Once the best prediction model is identified, the next logical research question was to examine how the prediction performance varies while limiting the number of features and sensors used. To evaluate this question, we utilize the random forest model since Table 6 showed that it had the highest mean accuracy, precision, recall, and F-Score when compared to the other classifiers. From this, a list of predictive/important features was established from most important to least important (see Figure 5) and a feature reduction was applied to have a more interpretable classifier. Table 7 reports the prediction results when features are limited to those from 25, 16, 13, 11, and 8. From the results in Table 7, one can see that the prediction performance does not vary significantly as the number of features’ is reduced. Some even match the RF performance utilizing all features (i.e., 43 features). Considering that the RF model presented a similar performance among 23, 16, and 11 features, their confusion matrix was analyzed to observed the prediction performance in two minority classes, i.e., very high fatigue and high fatigue states. These predictions are more interesting and more valuable since they are essential to avoid any risk in rehabilitation scenarios. The RF classifier presented fewer misclassifications between the low class and the two minority classes with 16 features illustrated in Figure 4b. Based on this observation, it is recommended to use the RF classifier with 16 features with an overall of 91.5% in all features. While the prediction performance is almost the same, the unnecessary features from the data were removed, optimizing computational costs and running time. Moreover, a smaller number of features facilitates the interpretation of the model, which is essential in the fatigue identification and diagnosis phases.

The prediction performance variation, while limiting the sensor used, is also reported in Table 6. Note that the sensor used to measure EMG is removed considering that EMG is invasive, which complicates its daily usage for real-time fatigue detection. Hence, the characteristics obtained by the EMG sensor were not considered. This means that the main features that detected fatigue were eliminated according to the sensor used. As expected, without EMG measurements the classifier presented a performance reduction since these features presented great importance in fatigue detection (see Figure 5). However, the classifier performance remains positive using two sensors (IMUS placed on footstep and around L2-S1) with an F-score of 88% and an overall average of 94%. Similarly, using one IMU located in the footstep has a performance of 84% and an overall average of 92%. Based on this observation, it suggests using only the IMU located on foot. While the prediction performance is almost the same, the costs incurred by the clinics are much lower, and the usability of the system by using only one sensor is significantly improved. These results are comparable with the studies that proposed fatigue estimation models during the walking task [69,71,73,75], which have shown accuracy values among 77% and 92%, with only two fatigue conditions—fatigued and non-fatigued. Besides, these studies [69,71,75] use motion capture systems as fatigue detection techniques that often require special setups, which makes them better suited for controlled environments than real rehabilitation scenarios.

Considering these observations, one can indicate three main contributions. First, the framework proposed has shown higher detection performance (with fewer features) and detects four fatigue diagnosis states in walking tasks, which allows clinicians to better monitor the patient and pinpoint the hazard, and to prescribe and manage interventions according to each individual’s needs. Second, the insights from the fatigue identification phase of our framework can be used to inform sensor placement and selection. Third, and more importantly, our framework presents a systematic approach that can answer the question: “what are the gains associated with wearing an extra sensor?” In essence, this question is left open to the researchers and practitioners to attempt to quantify whether the hassle and cost associated with wearing an extra sensor can be justified by a significant/practical improvement in fatigue detection when developing models for detecting/managing fatigue in other settings or their target application. The results and data used in this study can be accessed through the link provided in the Data Availability Statement Section. These codes can be used to develop predictive physical fatigue models.

The implementation of our proposed model requires an understanding of the specific features that were selected in the feature reduction process. Significant features contributing to the determination of physical fatigue in this model include:

- EMG RMS signals (features 41, 42, 39, 40) represent the square root of the average power of the EMG signal for a given period. Decrease over time of these signals led to the detection of muscle fatigue.
- Gait Acceleration Mean (feature 0) reflects the mean duration of each gait cycle. The fatigued musculoskeletal system is less able to attenuate heel strike-initiated shock waves, which could be observed as an increase in the amplitude of the acceleration measured at the foot. If the mean gait cycle time increased significantly with elapsed walking time indicates that the individual is fatigued.
- Gait Acceleration Median (feature 4) is the median value for each gait cycle acceleration.
- Spine Acceleration Mean (features 19 and 29) represents the torso acceleration over each gait cycle. These features show that if participants kept consistent torso movement over gait cycles, then it likely corresponds to their walking behavior and the patient is less likely to report physical fatigue.
- Spine Acceleration Median (features 33 and 23) is a measure of the central tendency of the torso acceleration distribution. Where the participant maintains a high level of spine acceleration, they are more likely to feel physically fatigued.
- Gait Maximum Acceleration (features 2 and 12) as the gait cycle time increased significantly with increasing fatigue, gait acceleration decreases. If the participant reduces their walking speed, then a decrease in peak gait acceleration is generated, indicating that the participant is fatigued.

The selected features for the best model for physical fatigue detection were shown in Figure 5. They are consistent with previous studies that have used IMUs for monitoring physical activity. Common features computed from the acceleration signal are the mean [68,94,95,98,100], variance or standard deviation [68,95,101,120] and the entropy and energy of the data [61,68].

Concerning the results using only one IMU located around the center of L5-S1, according to the literature review, it was expected to have a better classifier performance considering that the torso is the body part commonly used for physical fatigue detection and development models as reported in the following studies [38,71,73,74,75]. Specifically, Mamman et al. [38] reported that one IMU located on the torso was enough to detect fatigue in manual material handling environments. However, our results differ from the results obtained in previous studies. In this study we found that the mean back rotational position was selected as a mainly important feature for the classifier, as illustrated in Figure 5 with features numbers 19, 33, 29, and 23. The results showed that the accuracy and F-score were reduced to 88% and 66%, respectively, which is not promising. The above may suggest that (i) The features extracted from this sensor differ from other related studies; hence, the feature extraction methods influenced the classification performance, and should be improved. (ii) The use of a single sensor placed at L5-S1 in walking tasks may not be sufficient and consequently, the performance of the classifier decreases significantly. (iii) The fourth fatigue diagnoses states may increase the probability of failing in fatigue estimation with the use of this sensor in relation to only two fatigue diagnostic conditions that have been presented in the previous studies. Therefore, as future work, it is suggested to revise the obtained features to improve the performance of the classification model.

Similarly, we ound that the mean back rotational position was selected as the most important feature, as illustrated in Figure 5 with features numbers 19, 33, 29, and 23. Moreover, Mamman et al. [38] reported that an IMU located the torso was enough for detecting fatigue in manual material handling environments. However, the features utilized differ from the features extracted in this work. The above may suggest three things: (i) the feature extraction methods influenced the classification performance, and hence feature extraction with this sensor needs to be improved; (ii) the classifier performance decrease significantly with the use of only one sensor placed on L5-S1 in the walking tasks; and (iii) fourth fatigue diagnoses states may increase the probability of failing in fatigue estimation concerning only two fatigue diagnoses conditions. Therefore, as future work, it is suggested to review the characteristics obtained to improve the performance of the classification model.

On the other hand, there are a few limitations that must be acknowledged for this study. First, the sample size is small due to the confinement caused by the COVID-19 bio-safety protocols and the time required for each participant. However, the sample size is consistent with other studies that have focused on lab-based modeling of physical fatigue presented in Table 1. Second, the effect of different demographic variables (i.e., age, sex, physical condition) needs to be explored in future models of physical fatigue due to all volunteers being healthy people, and the features may show different behaviors and patterns with patients or other groups with various physical conditions. Third, the evaluation of our framework’s performance was limited to focused lab experiments. Future studies should evaluate how this framework performs in clinical scenarios.

Finally, a future and great application would be to implement this physical fatigue detection model in SAR systems. In essence, the system is proposed to be composed of a single-sensor interface, aiming to measure all features relevant to the RF classifier. The interface architecture comprises different nodes (e.g., sensor and SAR nodes) to adequately handle the difference in sampling rates and the amount of information acquired by the system. In the case of the sensor interface, the output of each node is the data processed, and for the the corresponding behavior and feedback of the SAR node. The data from the sensors would be stored in a database to be analyzed online to manage the interaction of the SAR with the user. Regarding the SAR’s behavior, the node should be designed considering three situations [81,121] (e.g., motivation, warning, and emergency, which are triggered depending on the data provided by the interface) while monitoring the users’ performance, with the aim of positively influencing the constant monitoring of user’s performance and providing feedback to motivate them while the physical exercise intensity and the correct exercise execution is controlled. Besides, SARs should be designed to communicate with the therapists if an emergency event occurs during therapy (e.g., physical fatigue above the maximum allowable level, dizziness or any parameter is out of normal). The aforementioned allows to achieve a successful rehabilitation program through continuous patient monitoring and providing feedback to clinicians about the patient’s performance to avoid overtraining, which can affect their rehabilitation and even induce physical suffering or physiological complications. It should be emphasized that this proposal is also applicable to other robot-assisted physical exercise scenarios, since this model allows for a close measurement of users’ physiological events.

## 6. Conclusions

Physical fatigue is a significant safety concern in rehabilitation environments and monitoring physical fatigue is essential to prevent an accident, injury occurrences, and for a rehabilitation program to be successful. The utilization of predictive models for physical fatigue modeling can provide a chance to understand the physiology and psychology of fatigue better. In this paper, an integrated framework was proposed with the main steps for developing a fatigue classification model using few features. It facilitates the interpretation model, which is essential in fatigue identification and diagnosis through visual analytical approaches allowing practitioners to identify risks which should be addressed through an appropriate intervention strategy.

The classifier models implemented considered four fatigue stages, instead of only determining whether the user is fatigued or not fatigued, as previous studies have presented, that allow to improve the fatigue monitoring and enable clinicians to prescribe an optimal therapeutic dose according to the individual’s needs, avoiding injuries and affectations to the process. The classifier is able to obtain greater accuracy, despite the fact that identifying the four fatigue states increases the probability of error. In particular, an accuracy of 96% and an F-score of 93% were obtained with random forest model, generating an optimal classifier.

The determination of the four fatigue states was achieved using blood lactate measurement. This parameter was the most used to measure performance and fatigue of individuals, thus being a direct indicator of fatigue. This in turn makes the method more accurate and reliable, since the data delivered by the sensors are directly related to what happens physiologically with the user.

The classifier performance model was also analyzed, checking the number of features and sensors used in future real-time applications. The dimensionality of data is vital to optimizing computational costs and running time. In addition, with fewer features, the total number of sensors required to estimate them could be reduced. The results showed that the classifier is capable of using a single sensor, maintaining an accuracy and F-score of 94% and 92%, respectively. Therefore, clinicians will also be more inclined to adopt the framework if it requires fewer sensors since it will: (i) be much cheaper, for example, requiring one or two IMUs instead of four, which would reduce the cost; (ii) make the process less invasive to the patient; and (iii) reduce the time needed for the patient to wear and strap on all the sensors.

## Figures and Tables

**Table 1:** A summary of fatigue modeling research.

Research	Physical Activity	Fatigue Detection Technique	Method
Maman et al. (2020) [38]	Manufacturing task	IMUs, HR, Borg Scale	SVM, RF, LR, PLR
Maman et al. (2017) [74]	Manufacturing task	IMUs, HR, Borg Scale	PLR
Zhang et al. (2014) [69]	Walking	3D optical tracking, IMUs	SVM
Karg et al. (2014) [70]	Squats	3D optical tracking, subjective scale	HMM, LR
Lee et al. (2009) [75]	Walking	3D optical tracking	LDA, Statistical test
Karg et al. (2008) [71]	Walking	3D optical tracking	LDA, SVM, kNN, NB
Helbostad et al. (2007) [76]	Walking	Accelerometers	Statistical test
Kavanagh et al. (2006) [72]	Walking	EMG	Statistical test
Yoshino et al. (2004) [73]	Walking	subjective scale, EMG, accelerometers	LR

SVM = support vector machines, RF = random forest, LR = logistic regression, PLR = penalized logistic regression, HMM = hidden markov models, LDA = linear discriminant analysis, kNN = k-nearest neighbors, NB = naive bayes.

**Table 2:** Summary of participants’ descriptive data (M ± SD). BMI, body mass index.

Gender	Age [YearsOld]	BMI [kg/m${}^{2}$]	Walking Speed [m/s]
Male	21.83 ± 1.40	22.84 ± 2.90	0.18 ± 0.37
Female	21.64 ± 0.74	22.25 ± 3.09	0.18 ± 0.35

**Table 3:** Borg scale description and classification.

Borg CR10 Value	Description	Classification
0	No exertion at all	Low
1	Very easy	
2	Easy	
3	Somewhat moderate	Moderate
4	Moderate	
5	Somewhat hard	
6	Hard	High
7	Very Hard	
8	Very Very Hard	
9	Extremely hard	Very High
10	Maximum exertion	

**Table 4:** Features generated.

N${}^{\circ }$	Feature	Description	Ref.
0	gait_mean_acce	Average gait acceleration	
1	gait_std_acce	Average gait acceleration std	
2	gait_max_acce	Average gait maximum acceleration	
3	gait_var_acce	Average gait acceleration variance	
4	gait_median_acce	Average median gait acceleration	
5	gait_energy_acce	Average gait acceleration energy	
6	gait_entropy_acce	Average gait acceleration entropy	
7	gait_kurtosis_acce	Average gait acceleration kurtosis	
8	gait_maxfreq_acce	Average gait acceleration maxfreq	
9	gait_stdfreq_acce	Average gait gyro stdfreq	
10	gait_mean_gyro	Average gait angular velocity mean	
11	gait_std_gyro	Average gait angular velocity std	
12	gait_max_gyro	Average gait maximum angular velocity	
13	gait_var_gyro	Average gait angular velocity variance	
14	gait_median_gyro	Average median gait angular velocity	
15	gait_energy_gyro	Average gait angular velocity energy	
16	gait_entropy_gyro	Average gait angular velocity entropy	
17	gait_curtosis_gyro	Average gait angular velocity kurtosis	
18	gait_maxfreq_gyro	Average gait angular velocity maxfreq	
19	l2_mean_acce	Average ts acceleration	[9,95]
20	l2_std_acce	Average ts acceleration std	[98,99]
21	l2_max_acce	Average ts maximum acceleration	[94,100]
22	l2_var_acce	Average ts acceleration variance	[101,102]
23	l2_median_acce	Average ts acceleration velocity	[76,103]
24	l2_energy_acce	Average median ts acceleration energy	[70].
25	l2_entropy_acce	Average ts acceleration entropy	
26	l2_kurtosis_acce	Average ts acceleration kurtosis	
27	l2_maxfreq_acce	Average ts acceleration maxfreq	
28	l2_stdfreq_acce	Average ts acceleration maxfreq std	
29	l2_mean_gyro	Average ts angular velocity mean	
30	l2_std_gyro	Average ts angular velocity std	
31	l2_max_gyro	Average ts maximum angular velocity	
32	l2_var_gyro	Average ts angular velocity variance	
33	l2_median_gyro	Average median ts angular velocity	
34	l2_energy_gyro	Average ts angular velocity energy	
35	l2_entropy_gyro	Average ts angular velocity entropy	
36	l2_Kurtosis_gyro	Average ts angular velocity kurtosis	
37	l2_maxfreq_gyro	Average angular velocity maxfreq	
38	l2_stdfreq_gyro	Average ts angular velocity maxfreq std	
39	rms_gastro	RMS envelope of the gastrocnemius signal	
40	rms_tibilisAnterior	RMS envelope of the tibilis anterior signal	
41	rms_rectusFemoris	RMS envelope of the rectus femoris signal	
42	rms_bicepsFemoris	RMSenvelope of the biceps femoris signal	

Ref. = References; ts = torso swing; std = standard deviation; Maxfreq = maximun frequency; stdfreq = frequency standard deviation; RMS = root-mean-square.

**Table 5:** Data distribution in the dataset according to each fatigue state.

Class	Number of Samples
Very High	463 (15.86%)
High	732 (25.08%)
Moderate	771 (26.41%)
Low	953 (32.65%)
Total	**2919 (100%)**

**Table 6:** Mean performance of the classification methods for fatigue detection in walking task. Bold values show the best score for each performance metric.

Model	Hyperparameters	Accuracy	Precision	Recall	F1-Score
**RF**	**estimators = 100**	**0.965**	**0.931**	**0.929**	**0.928**
ANN	activation = tanh solver = adam HLS = (100, 100, 100) alpha = 0.0001 learning_rate = ‘constant’ max_iter = 1000	0.949	0.896	0.898	0.894
SVM	kernel = rbf class_weight = ‘balanced’ C = 64	0.907	0.809	0.809	0.806
DT	criterion = entropy max depth = 12 min samples split = 11 min samples leaf = 4	0.907	0.806	0.805	0.804
KNN	neighbors = 3	0.908	0.807	0.805	0.804
LR	solver = newton-cg C = 1,000,000	0.822	0.626	0.624	0.620

**Table 7:** Mean performance of the random forest model for fatigue detection in walking task using feature reduction and different sensors combinations. The best performance model is in **bold**.

Sensors	Estimators	Features	Accuracy	Precision	Recall	F1-Score
	60	25	0.965	0.934	0.928	0.930
Thigh (EMG),	**40**	**16**	**0.965**	**0.932**	**0.927**	**0.929**
Shank (EMG),	80	13	0960	0921	0916	0917
L5-S1, Foot	80	11	0963	0.926	0.925	0.925
	100	8	0946	0.895	0.883	0.888
L5-S1, Foot	80	17	0.940	0.883	0.876	0.879
L5-S1	80	16	0.839	0678	0.658	0.664
Foot	80	19	0921	0.856	0.827	0.838

## Data Availability

Publicly available datasets were analyzed in this study. This data can be found here: https://figshare.com/projects/Fourth_fatigue_diagnosis_states/119580 (accessed on 12 September 2021).
